# Alterations, Interactions, and Diagnostic Potential of Gut Bacteria and Viruses in Colorectal Cancer

**DOI:** 10.3389/fcimb.2021.657867

**Published:** 2021-07-06

**Authors:** Renyuan Gao, Yefei Zhu, Cheng Kong, Kai Xia, Hao Li, Yin Zhu, Xiaohui Zhang, Yongqiang Liu, Hui Zhong, Rong Yang, Chunqiu Chen, Nan Qin, Huanlong Qin

**Affiliations:** ^1^ Diagnostic and Treatment Center for Refractory Diseases of Abdomen Surgery, Shanghai Tenth People’s Hospital, Tongji University School of Medicine, Shanghai, China; ^2^ Institute for Intestinal Diseases, Tongji University School of Medicine, Shanghai, China; ^3^ Department of General Surgery, Shanghai Tenth People’s Hospital, Tongji University School of Medicine, Shanghai, China; ^4^ Department of Pediatrics, Shanghai Tenth People’s Hospital, Tongji University School of Medicine, Shanghai, China

**Keywords:** gut microbiome, bacteria, virus, bacteriophage, microbial network, diagnostic test

## Abstract

Gut microbiome alteration was closely associated with colorectal cancer (CRC). Previous studies had demonstrated the bacteria composition changes but lacked virome profiles, trans-kindom interactions, and reliable diagnostic model explorations in CRC. Hence, we performed metagenomic sequencing to investigate the gut microbiome and microbial interactions in adenoma and CRC patients. We found the decreased microbial diversity in CRC and revealed the taxonomic alterations of bacteria and viruses were highly associated with CRC at the species level. The relative abundance of oral-derived species, such as *Fusobacterium nucleatum*, *Fusobacterium hwasookii*, *Porphyromonas gingivalis*, and *Bacteroides fragilis*, increased. At the same time, butyrate-producing and anti-inflammatory microbes decreased in adenoma and CRC by non-parametric Kruskal-Wallis test. Despite that, the relative abundance of *Escherichia viruses* and *Salmonella viruses* increased, whereas some phages, including *Enterobacteria phages* and *Uncultured crAssphage*, decreased along with CRC development. Gut bacteria was negatively associated with viruses in CRC and healthy control by correlation analysis (P=0.017 and 0.002, respectively). Viruses were much more dynamic than the bacteria as the disease progressed, and the altered microbial interactions were distinctively stage-dependent. The degree centrality of microbial interactions decreased while closeness centrality increased along with the adenoma to cancer development. *Uncultured crAssphage* was the key bacteriophage that enriched in healthy controls and positively associated with butyrate-producing bacteria. Diagnostic tests based on bacteria by random forest confirmed in independent cohorts showed better performance than viruses for CRC. In conclusion, our study revealed the novel CRC-associated bacteria and viruses that exhibited specific differences and intensive microbial correlations, which provided a reliable diagnostic panel for CRC.

## Introduction

Colorectal cancer (CRC) is a malignant tumor that occurs in the colon and rectum and is a severe threat to public health worldwide (Luebeck and Moolgavkar, 2002; Chen et al., 2016; Siegel et al., 2017). Development of CRC is a multi-step event that is influenced by host genetics as well as various environmental factors, including geography, diet, and gut microbiota (Park et al., 2009; Tjalsma et al., 2012; Yatsunenko et al., 2012; Jiao et al., 2014; Gallego et al., 2015; Hsu et al., 2015; Usher-Smith et al., 2016). Gut microbiota is a highly sophisticated community that comprises bacteria overwhelmingly and viruses, fungi, and archaebacteria. This relatively stable conglomerate is involved in various essential functions of the immune system and metabolism of the host (Lim et al., 2016). Alterations of the gut microbiome, on the other hand, are detrimental to the host and have been reported in many chronic diseases, including CRC (Huycke et al., 2002; O’Keefe, 2016).

Moreover, aberrant enteric microbial communities coincide with the disease progression of CRC (Thomas et al., 2019). The latest evidence has shed light on the biological roles of several prominent gut microbes in the development of this gastrointestinal (GI) cancer. For example, *Fusobacterium nucleatum*, enriched in CRC patients (Castellarin et al., 2012; Yu et al., 2017a), invades the colon epithelial cells through the damaged gut barriers *via* FadA adhesion, Fap2, or an altered immune microenvironment (Kostic et al., 2013; Rubinstein et al., 2013; Gur et al., 2015). *Fusobacterium nucleatum* interacts with specific Toll-like receptors in the tumor cell, thereby triggering intracellular signaling, inducing gene mutation, and promoting tumorigenesis (Mima et al., 2015; Mima et al., 2016; Yang et al., 2017). Another CRC-enriched bacterium, *Peptostreptococcus anaerobius*, contributes to the carcinogenesis of CRC by modulating cholesterol biosynthesis (Tsoi et al., 2017). Also, the altered gut microbes correlate with gene mutations involved in the CRC. *Fusobacterium nucleatum* is confirmed to be associated with MSI-high status (Mima et al., 2015; Mima et al., 2016).

In comparison with gut bacteria, relatively little is known regarding the involvement of their viral counterparts in CRC (Hannigan et al., 2018; Nakatsu et al., 2018), which is, in part, attributed to the latter’s relatively low abundance. Nevertheless, human viruses are closely linked to the occurrence of many cancers, such as hepatitis and hepatic carcinoma, nasopharynx cancer, cervical cancer, and GI cancers (Dahiya and Renuka, 2017; Tu et al., 2017; Chen et al., 2019; Cohen et al., 2019). Another major group of gut viruses is bacteriophages, which can significantly affect their prokaryotic hosts and influence disease development (Dahiya and Renuka, 2017). Current evidence has unraveled that bacteria and viruses are closely associated with many diseases, especially cancers. Whereas studies so far on gut microbiome traits of CRC have revealed multiple gut microbial features of cancer, they either focused on bacteria or analyzed microbes in separate kingdoms (e.g., Bacteria, Virus, Fungus), which overlook the intrinsic connections between members of the major phylogenetic branches (Coker et al., 2019).

This study examined the taxonomic and functional alterations of gut microbiota, microbial interactions within bacteria and viruses in individuals with colonic adenoma and CRC. Our results provided a comprehensive overview of microbial compositional alteration, bacteria, and viruses interactions in CRC development and CRC-associated microbes for CRC diagnostic.

## Materials and Methods

### Enrollment of CRC Patients, Adenoma Patients, and Healthy Controls

We enrolled patients with CRC and colonic adenoma in Shanghai Tenth People’s Hospital between 2015 and 2018. Individuals suspected of colorectal cancer due to rectal bleeding, abdominal pain, and change of defection habit or asymptomatic individuals undergoing colonoscopy in the clinic were enrolled and asked to provide stool samples and then confirmed by colonoscopy and pathology. The exclusion criteria included exposure to antibiotics within one month, a history of gastrointestinal surgery, probiotics or prebiotic consumption within 1 month, diagnosed with acute or chronic diarrhea and hepatitis. Correspondingly, a total of 71 CRC patients and 63 adenoma patients were included in this study. Also, 91 healthy individuals matched in age, BMI, and gender ratio were recruited as controls (**Supplementary Table 1**). The Ethics Committee of Shanghai Tenth People’s Hospital had approved this study protocol. Informed consent was obtained from all patients. All procedures were performed following the Declaration of Helsinki and its later amendments.

### Sample Collection and DNA Extraction

Fresh fecal samples were collected from the recruited subjects and were transported to the laboratory with an ice pack within two hours. All samples were then frozen immediately and stored at - 80°C before analyses.

According to the manufacturer’s instructions, QIAamp Fast DNA Stool Mini Kit (Qiagen, Hilden, Germany) was used for DNA extraction. Briefly, 20 μl proteinase K solution (20 mg/ml) and 100 mg zirconium beads (0.1 mm) were added to the fecal samples before the mixture was fully homogenized on a Mini-Beadbeater (FastPrep, Thermo Electron Corporation, USA) and then supplemented with buffer AL. The resulting mixture was incubated at 70°C for 10 min and supplemented with 200 μl ethanol (96%). Then the mixture to the QIAamp spin column and centrifuged at 8000 g for 1 min. The column was washed successively with 500 μl buffer AW1 and 500 μl buffer AW2. Finally, DNA was eluted with 100 μl buffer AE. The DNA concentration was measured using a NanoDrop (Thermofisher, USA). The integrity and size of the extracted DNA were examined with electrophoresis on 1% agarose gel containing 0.5 μg/ml ethidium bromide.

### Shotgun Metagenomic Sequencing

For Illumina library preparation, genomic DNA was sheared to an average 500-bp fragment length using a Bioruptar ultrasonicator. Following the Illumina TruSeq DNA Sample Prep v2 Guide (Illumina, Inc., San Diego, CA, USA), DNA paired-end libraries were constructed for the 225 fecal samples (71 from CRC patients, 63 from colorectal adenoma patients, and 91 from normal controls). The quality of all libraries was evaluated using an Agilent bioanalyzer (Agilent Technologies, Wokingham, UK) and the DNA LabChip 1000 kit. All samples were subject to 150 bp paired-end sequencing on an Illumina HiSeq platform (Illumina, Inc., San Diego, CA, USA).

Raw reads were pre-processed for 3’ end trimming using a quality threshold of 30 and filtered to exclude adaptor contaminated reads and low-quality reads (e.g., reads containing more than 50% nucleotides below Q30, reads short than 70 bp, and reads mapped to the human genome based on alignment with SOAPaligner 2.21) (Li et al., 2008). Consequently, an average of 93.3% of high-quality reads (defined as clean data) was obtained from all samples.

### *De Novo* Assembly and Construction of the Gene Catalog

To construct the gut gene catalog, SOAPdenovo (version 2.04) was used to assemble high-quality reads from each sample into contigs (Luo et al., 2012; Gao et al., 2020). MetaGeneMark (version 3.26) was used to predict open reading frames (ORFs) in contigs (Noguchi et al., 2006). To obtain a non-redundant gene set, a pairwise comparison of predicted ORFs (filtered with a length of 100 bp) was performed using CD-HIT (version 4.5.7) at 95% identity and 90% coverage (Li and Godzik, 2006). The resulting non-redundant gene catalog contained 4,071,018 microbial genes, which had an average length of 786 base pairs. Functional annotations were carried out by BLASTP search against the KEGG database (e value ≤ 1e − 5 and high-scoring segment pair scoring > 60) (Kanehisa et al., 2004). For each functional feature (KO in the KEGG database), we estimated its abundance by accumulating the relative abundances of all affiliated genes. Samples were functionally profiled using HUMAnN2, which uses the MetaCyc pathway database and MinPath to identify a parsimonious set of pathways explaining observed reactions in the community (Ye and Doak, 2009; Abubucker et al., 2012; Caspi et al., 2018).

### Taxonomic and Gene Profiling

Metagenomic reads were assigned to microbial taxa using the k-mer based algorithms as implemented in the Kraken taxonomic annotation pipeline (Wood and Salzberg, 2014).

For gene abundance profiling, SOAPalign2.21 was used to align clean reads to gene sequences. The gene relative abundance profile was generated following the procedure described by Qin et al. (2014). Reads aligned to multiple genes were allocated proportionally to read counts uniquely mapped to these genes (normalized by gene length).

### Gene Mutation Detection

The method of KRAS gene mutation had been described before (Gao et al., 2017). In brief, the common somatic mutations in exons 2, 3, and 4 of the KRAS gene were determined in the tissue samples using the AmoyDx KRAS Mutation.

Detection Kit (AmoyDx, Xiamen, China). All analyses were performed according to the manufacturer’s instructions. Extracted DNA was diluted and then transferred to a polymerase chain reaction (PCR) tube, and a real-time PCR (ABI 7500, Applied Biosystems, Foster City, CA, USA) was immediately performed. KRAS mutation status was determined according to the cut-off values defined in the kit instructions.

### Classifier

Random forest model (randomForest package in R) was used to build the classifier based on the abundance profiling of species abundance. The predictive performance (estimated by 10-fold cross-validation) was optimized by selecting species that displayed the best discriminatory power. The ROC curve was plotted using the pROC R package.

### Correlation Network

SparCC was used to construct the association network of gut microorganisms (Friedman and Alm, 2012). Briefly, bootstrapping of 1000 repetitions was used to compute the P-value for each correlation. The resulting two-sided P values<0.05 were shown in the network, which was visualized with Cytoscape3.0.2. We used the degree centrality, betweenness centrality, and closeness centrality to describe each node’s importance (Sadria et al., 2019).

### Statistical Analysis

The non-parametric Wilcoxon test/Kruskal-Wallis test (Wilcox. test/Kruskal.test in R) was employed to analyze the statistical significance of the gene, KO, enzyme, and different taxonomic levels (phylum, genus, and species). The relative abundance of these features was subjected to statistical analyses. The Benjamini-Hochberg method was used for correction in multiple comparisons in which an FDR (false discovery rate) value < 0.05 was considered significant. Enriched features with an adjusted FDR < 0.05 were identified, and the enrichment group was then determined according to a higher rank-sum value.

### Data Access

The metagenomic sequence data sets have been deposited in the NCBI Sequence Read Archive (SRA) with access numbers PRJNA706060 and PRJNA514108.

## Results

### Altered Gut Microbiome Architectures in Adenoma and CRC

**Figure 1:**
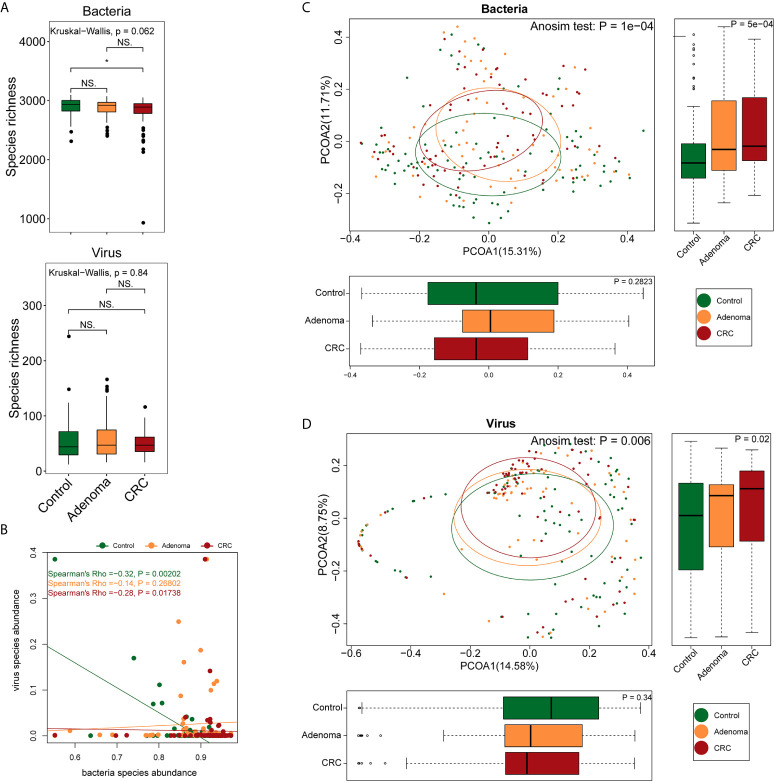
Differences in gut microbiome structure among the control (colored in green), adenoma (colored in amber), and CRC (colored in dubonnet) groups. **(A)** Comparison of bacterial or viral richness (*P < 0.05). **(B)** Association of bacteria and viruses. **(C)** Principal coordinate analysis of the bacteria. **(D)** Principal coordinate analysis of the viruses. NS, Not significant.

A total of 2,136 Gb clean data were generated, yielding an average of 9.49 ± 3.74 Gb per sample (92.58%). Bacterial, viral, and fungal reads accounted for 97.2%, 2.16%, and 0.58% of the total clean data and were mapped to 3,184 bacterial, 1,199 viral, and 1,132 fungal species, respectively. The healthy controls, adenoma, and CRC groups shared approximately 99% of the bacteria and 39.4% (473) of the viruses. The bacteria richness of the CRC group was lower than that of the control group (P=0.024, Wilcoxon signed-rank test) (**Figure 1A**). No differences were observed either in viral richness or Shannon or Simpson indexes among the three groups. As consistent with a previous report (Nakatsu et al., 2018), there was a significant negative correlation between viruses and bacteria in the healthy control and CRC groups but not in the adenoma group (**Figure 1B**). Despite the overall comparable microbial diversity, the three groups exhibited apparent differences in microbial structure (Anosim P=1*10^-4^ for CRC *vs*. healthy control and P=0.0026 for adenoma *vs*. healthy control, **Figures 1C, D**).

**Figure 2:**
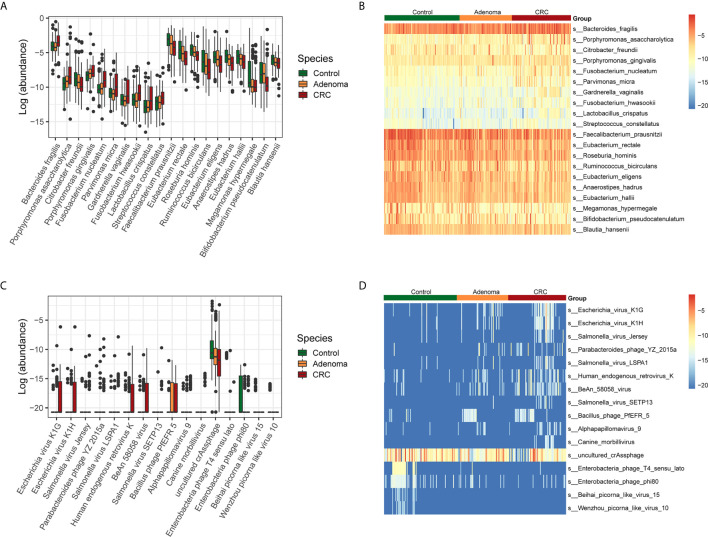
Relative abundances of bacterial and viral species in control (colored in green), adenoma (colored in amber), and CRC (colored in dubonnet) groups. **(A)** Top 10 bacteria showed increased or decreased relative abundance in the CRC group than in the control group. The vertical axis represents relative abundance. **(B)** Heatmap of the top 10 bacteria that showed increased (top 10) or decreased (lower 10) relative abundance in the CRC group compared with that in the control group. **(C)** The 15 viruses that were different in relative abundance between the control and CRC groups, including 11 enriched and top 4 depleted in the CRC group. **(D)** Heatmap of the 15 viruses that were different in relative abundance between the control and CRC groups.

Next, we examined the variations of bacterial and viral species among the three groups (**Supplementary Figures 1A–C**). The taxonomic alteration at the phylum level showed that only Fusobacteria was significantly different in the three groups (0.10% ± 0.26%, healthy controls *vs*. 0.99% ± 6.86%, adenoma group *vs*. 0.39% ± 1.44%, CRC group, P=9.78*10^-4^, FDR=0.03). The top ten bacteria that increased and decreased from adenoma to CRC were displayed (**Figures 2A, B**). Besides, the top ten viruses exhibiting increased and decreased relative abundance from adenoma to CRC were also showed (**Figures 2C, D**).

Then we identified the potential microbes that enriched in CRC when compared with healthy control by LEfSe. Similar to the above results, nine bacteria, including *Bacteroides fragilis*, *Escherichia coli*, *Porphyromonas asaccharolytica*, *Prevotella intermedia*, *Fusobacterium varium*, *Porphyromonas gingivalis*, *Fusobacterium nucleatum*, *Citrobacter freundii*, and *Parvimonas micra*, dominated in the CRC group. In contrast, 28 beneficial bacteria, such as *Faecalibacterium prausnitzii*, *Eubacterium rectale*, *Roseburia hominis*, as well as some *Bifidobacterium* species, dominated the HC groups (**Supplementary Figure 2A**). In addition, we also found that *Suid_alphaherpesvirus_1* dominated in the CRC samples, *Bacteroides_phage_B124_14*, and *Escherichia_virus_VR26* dominated in the adenoma group while *uncultured_crAssphage* and *Enterobacteria_phage_T4_sensu_lato* dominated in the healthy control group (**Supplementary Figure 2B**).

### Bacteria and Virus Associated With Tumor Stages and Gene Mutations

Since CRC development comprises multiple stages and involves dozen gene mutations, which affected the prognosis of CRC, it was essential to unravel the potential gut microbes associated with tumor stages and gene mutations. We compared the gut bacteria and viruses of CRC patients by early (TNM stage I and II) and later (TNM stage III and IV) stages. By comparing the relative abundance of these viruses, we found that only *BeAn_58058_virus*, *Escherichia_virus_K1G*, and *Escherichia_virus_K1H* were the three significant enriched viruses regardless of early-stage or later stage in comparison with healthy control (all FDR<0.05). Then we compared the early and later stages of CRC and found that bacteria *Klebsiella* species such as *Klebsiella quasipneumoniae, Klebsiella oxytoca*, and *Klebsiella variicola* and virus *Klebsiella_virus_PKP126* dominated at the early stage of CRC while bacteria *Faecalibacterium prausnitzii*, *Bacillus cereus*, and *Lactococcus species* and *virus Bacillus_phage_PfEFR_5* dominated at the later stage of CRC (**Supplementary Figures 2C, D**).

Then we also compared the gut microbiota of CRC patients with or without KRAS gene mutation and the status of microsatellite stability (MSI or MSS). We found that KRAS gene mutation was associated with *Parabacteroides_phage_YZ_2015a* enrichment, while the wild type KRAS was associated with *Pseudomonas_phage_phiKZ* enrichment (**Supplementary Figure 3A**). Bacteria *Lachnoclostridium phocaeense*, *Bifidobacterium breve*, and several phages such as *Bacillus_phage_PfEFR_5*, *Uncultured_phage_WW_nAnB_strain_2*, *Enterobacteria_phage_mEpX2* were found enriched in the CRC patients with MSI (**Supplementary Figures 3B, C**).

### Dynamic Microbial Networks Within and Between Bacteria and Virome in CRC

We further assessed the relationships within or between bacteriome and virome in the CRC, adenoma, and HC groups (**Supplementary Figures 4A, B**). The astringent criterion was applied to study the most robust inter-microbial interactions. The bacterial network showed that Fusobacteria *species*, *Porphyromonas asaccharolytica*, *Parvimonas micra* were the significant microbes of CRC that correlated with *Porphyromonas gingivalis* in the adenoma group and negatively associated with *Bifidobacterium* species in the healthy controls. Regarding the viral network, nine viruses positively correlated with each other, while four were negatively associated with *Bacillus_phage_PfEFR_5* in adenoma. Several phages dominated the healthy control, including *uncultured_crAssphage* and *Enterobacteria_phage_phi80*, which were negatively associated with viruses in the CRC group.

**Figure 3:**
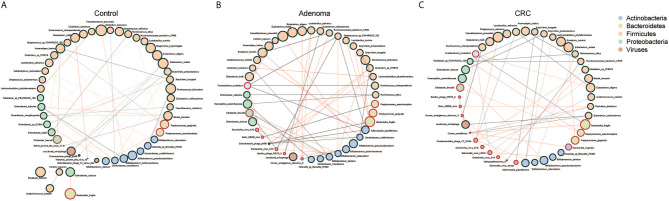
Co-occurrence networks of the marker species in the control **(A)**, adenoma **(B)**, and CRC **(C)** groups. Each microbial species is marked by its affiliating phylum, which is shown in the top right. A microbial species and cooccurrence relationship are indicated by a node and an edge, respectively. A connection (line between dots) indicates a significant (FDR < 0.05) correlation. The size of each node is proportional to the relative abundance of the species. Lines between nodes indicate positive correlations (green) or negative correlations (red).

Then we evaluated the network between bacteria and viruses in three different groups. Three centrality indicators were used to describe the importance of gut microbes in the network (Jiao et al., 2017). Betweenness centrality indicated the potential influence of a node on other nodes. Closeness centrality indicated a node closest to other nodes in the network, reflecting the information transmission speed between nodes. Degree centrality indicated a specific node had the highest number of communication with other nodes. The betweenness centrality of the gut microbiome was significantly higher in control than CRC (**Supplementary Figure 5A**). The Closeness centrality increased while Degree centrality decreased significantly from adenoma to CRC (**Supplementary Figures 5B, C**). We found that bacteria and viruses showed a negative association generally with significant differences only in CRC and HC, not in adenoma (**Figure 2B**). Of all the three groups, species of Firmicutes dominated while those of Actinobacteria and Proteobacteria showed a comparable proportion. Several species, such as *Faecalibacterium prausnitzii*, *Eubacterium hallii*, *Porphyromonas gingivalis*, and *Porphyromonas asaccharolytica*, were all shared in the three networks (**Figure 3**). However, we also identified 4 and 10 species that dominated in the CRC group showed up in healthy control and adenoma groups. In addition, the *Fusobacterium nucleatum* only showed connections with other species in adenoma and CRC. *Bacteroides fragilis* was involved in all three groups but only positively connected with *Fusobacterium nucleatum* in CRC. We also revealed that the number of viruses involved in the network increased as the diseases progressed (5 in HC, 7 in adenoma, and 10 in CRC). Among them, only the *uncultured_crAssphage* dominated all three groups. Interestingly, at different disease stages, *uncultured_crAssphage* had various connections with certain bacteria. In the healthy control group, *uncultured_crAssphage* was positively associated with *Eubacterium rectale* (r=0.058, P=0.046) and *Butyrivibrio proteoclasticus*(r=0.068,P=0.015), but negatively correlated to *Bifidobacterium kashiwanohense* (r=-0.078,P=0.019) and *Lactobacillus salivarius* (r=-0.080,P=0.019) in adenoma. In the CRC, *uncultured_crAssphage* was positively associated with *Eubacterium cellulosolvens* (r=0.082, P=0.016). Finally, we estimated the relationships of bacteria and viruses in all samples (**Supplementary Figure 6**). Several CRC-related bacteria such as *Porphyromona species*, *Fusobacterium species*, *Streptococcus constellatus*, and *Parvimonas micra* were significantly correlated *BeAn_58058_virus*, *Human_endogenous_retrovirus_K*, and *Bacillus_phage_PfEFR_5*. We also observed that some species belong to phylum Firmicutes (*Thermoanaerobacterium_sp_RBIITD*, *Staphylococcus_muscae*, genus *Caldicellulosiruptor*, etc.) and Proteobacteria (*Acinetobacter soli*, *Shewanella frigidimarina*, *Campylobacter cuniculorum*, etc.) were positively associated with *Enterobacteria phage phi80*, *Picorna like virus*, and *Enterobacteria phage T4 sensu lato* but negatively associated with *Salmonella* and *Escherichia virus*. Different and complicated networks within bacteria, viruses, or between them were showed up at different stages (healthy control, adenoma, and CRC), indicating that the microbial networks were dynamic and evolving from adenoma to CRC.

### Gut Microbes That Contributed to the Alteration of Microbial Functions in CRC

**Figure 4:**
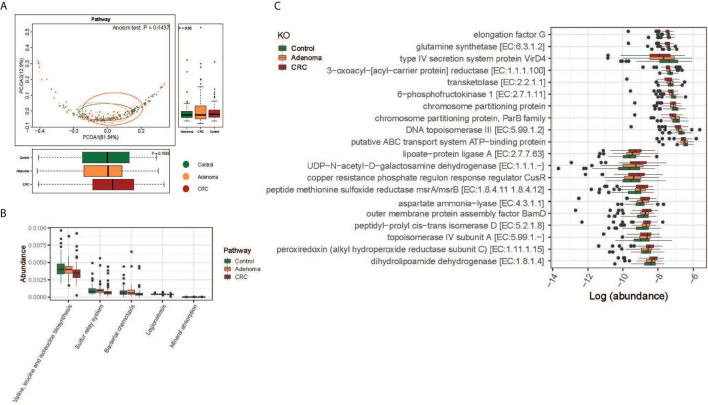
Functional differences of gut microbiome among the control (colored in green), adenoma (colored in amber), and CRC (colored in dubonnet) groups. **(A)** Principal coordinate analysis of the KEGG pathways. **(B)** The pathways that were different in relative abundance between the control and CRC groups. **(C)** The top 10 KOs that showed increased (top 10) or decreased (lower 10) relative abundance in the CRC group compared with that in the control group.

The functions of gut microbiota along the carcinogenesis sequence were also evaluated at various levels. No significant difference was observed at the pathway level among the three groups, nor between adenoma and healthy control at KO level(**Figure 4A** and **Supplementary Figure 5D**). But we detected significant differences in the functional structure of the community between CRC and controls at the pathway level (Anosim P=0.04; **Supplementary Figure 5E**). Relative abundances of different pathways were also displayed. And genes related to valine, leucine, and isoleucine biosynthesis (FDR=0.016), sulfur relay system (FDR=0.047), and mineral absorption (FDR=0.047) decreased along with the carcinogenesis (**Figure 4B**). Specifically, the top 10 decreased and increased microbial functions were showed at the KO level (**Figure 4C**).

### Diagnostic Ability of Bacteria and Virome in CRC

**Figure 5:**
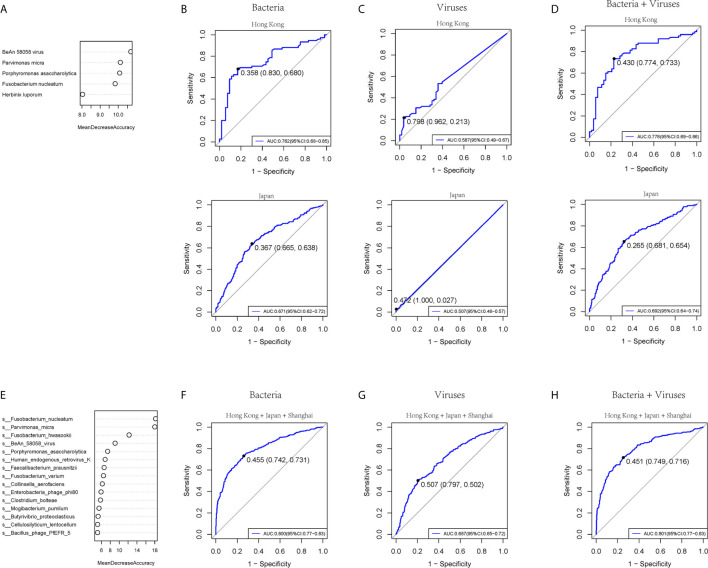
The prediction ability of bacterial and viral markers for colorectal cancer. **(A)** Importance ranking of the five bacterial or viral species based on random forest analysis. **(B)** The prediction performance of the five bacterial markers for the validation cohort of Hong Kong (upper panel) or Japan (lower panel). **(C)** The prediction performance of 5 viral markers for the validation cohort of Hong Kong (upper panel) or Japan (lower panel). **(D)** The prediction performance of 5 bacterial and five viral markers for the validation cohort of (upper panel) or Japan (lower panel). **(E)** Importance ranking of 15 bacterial and viral species based on random forest analysis across Hong Kong, Japan, and Shanghai cohorts. **(F)** The prediction performance of 15 bacterial markers for the three cohorts. **(G)** The prediction performance of 15 viral markers for the three cohorts. **(H)** The prediction performance of a 15-component panel of bacteria and viruses for the three cohorts.

To test the diagnostic ability of bacteria and virome in CRC, five bacteria and five viruses, as well as five markers including four bacteria and one virus based on the random forest, were chosen respectively in our cohort and tested in the published Hong Kong and Japanese cohorts of colorectal cancer patients (Yu et al., 2017a) (**Figure 5A** and **Supplementary Figure 7A**). The AUROC values of the bacteria model in the training, Hong Kong, and Japanese cohorts were 0.860 (95% CI: 0.80-0.92), 0.762 (95% CI: 0.68-0.85), and 0.671 (95% CI: 0.62-0.72) (**Figure 5B**). The viruses model showed that the AUROC values for distinguishing CRC from controls were 0.757 (95% CI: 0.68-0.83), 0.587 (95% CI: 0.49-0.67), and 0.507 (95% CI: 0.48-0.57) in the training, Hong Kong, and Japan cohort, respectively (**Figure 5C**). Five combined markers, including four bacteria and one virus, showed that the values of AUROC were 0.868 (95% CI: 0.81-0.93), 0.778 (95% CI: 0.69-0.86), and 0.692 (95% CI: 0.64-0.74) in the training cohort, Hong Kong and Japan cohorts (**Figure 5D**). We then combined the three cohorts from three different sites and chose the top 15 markers, including bacteria and viruses (**Figure 5E** and **Supplementary Figure 7B**). The values of AUROC of bacteria, viruses and combined bacteria and viruses were 0.800 (95% CI:0.65-0.72) (**Figure 5F**), 0.687 (95 %CI:0.77-0.83) (**Figure 5G**) and 0.801 (0.77-0.83) (**Figure 5H**). The evidence in the present study indicated that the bacteria model had better diagnostic efficacy than viruses within a limited species number for clinical application.

## Discussion

To our knowledge, this study presented the most comprehensive exploration of the gut microbiome in adenoma and colorectal cancer sequence so far. We revealed the bacteriome and virome profiles of CRC and their dynamic relationships at different stages. We also established the diagnostic models based on gut bacteria and virome and validated the accuracy and application in independent cohorts for colorectal cancer detection.

The betweenness and degree centrality decreased while closeness centrality increased in colorectal cancer, indicating the loose microbial architecture and fewer interactions as the disease progressed. We also showed a significant negative relationship between bacterial and viral species abundance in healthy control and CRC, but not in adenoma. This negative correlation was also observed between bacteria and bacteriophage diversity in CRC, which was in line with a previous study (Nakatsu et al., 2018). However, the conclusions needed to be confirmed in a larger cohort.

The present study identified several CRC associated bacteria, such as *Fusobacterium nucleatum*, *Bacteroides fragilis*, *Porphyromonas gingivalis*, and *Parvimonas micra* based on metagenomic sequencing, which were in line with previous studies (Lopez-Siles et al., 2016; Quevrain et al., 2016; Munukka et al., 2017; Yang et al., 2017; Yu et al., 2017a; Brennan and Garrett, 2019). The mechanisms study of these bacteria provided a causality relationship with CRC. *Fusobacterium nucleatum* promoted CRC through the interactions between fusobacterial lectin Fap2 and host polysaccharide and NK cells receptor TIGHT or adhesion between FadA and E-cadherin, as well as modulations of the tumor-associated immune cell and pro-inflammatory microenvironment (Kostic et al., 2013; Rubinstein et al., 2013; Gur et al., 2015; Abed et al., 2016; Ye et al., 2017). *Fusobacterium nucleatum* facilitated the carcinogenesis by modulating Wnt/b-catenin and NF-κB signal pathways (Yang et al., 2017; Rubinstein et al., 2019). *Fusobacterium nucleatum* was also found to induce chemotherapy resistance by BIRC3 expression and autophagy and promote CRC metastasis through long non-coding RNA Keratin7-antisense (KRT7-AS) and Keratin7 (Yu et al., 2017b; Zhang et al., 2019; Chen et al., 2020). *Bacteroides fragilis* was found to induce CRC by secretion of a 20 kDa metalloprotease toxin and activation of c-Myc expression and production of pro-inflammatory cytokines such as IL-17 and IL-8, as well as the RHEB/mTOR pathway (Sears, 2001; Wu et al., 2003; Hwang et al., 2013; Geis et al., 2015; Chung et al., 2018; Bao et al., 2019). *Porphyromonas gingivalis* promoted CRC by activating the MAPK/ERK signaling pathway (Mu et al., 2020). However, the potential role of *Parvimonas micra* in CRC was still underestimated.

To address the spatial-temporal change of gut microbes during the development of CRC, several models were proposed. One of the most arrestive hypotheses was the driver-passenger model in CRC, indicating that bacteria of driver role initiated the carcinogenesis and then disappeared accompanied by enrichment of bacteria with passenger role to continue the carcinogenetic process (Tjalsma et al., 2012). The present study showed the taxonomic alterations of specific microbes, such as *Bacteroides fragilis*, *Porphyromonas asaccharolytica*, *Fusobacterium nucleatum*, *Parvimonas micra*, *Fusobacterium hwasookii*, and *Streptococcus constellatus*, that increased along the stages of CRC. This was in line with our previous study, which showed the dominant bacteria in colorectal cancer tissues, indicating their role as passengers in CRC development (Sobhani et al., 2013; Gao et al., 2017). We also noticed that some bacteria, such as *Porphyromonas gingivalis*, *Escherichia coli*, and *Proteus mirabilis*, were enriched in the adenoma group compared to the CRC group, suggesting their potential function as driver microbes. However, this hypothesis still needed more evidence.

We demonstrated the explicit dysbiotic profiles of CRC, characterized by enrichment of bacteria, and unraveled the altered viral community in the gut. We identified that *Escherichia* and *Salmonella viruses* dominated in the CRC, while *uncultured_crAssphage* and *Enterobacteria phages* dominated healthy control. The relative abundance of *CrAssphage* also decreased from adenoma to CRC, indicating that this bacteriophage might play an essential role in inhibiting the carcinogenesis progress. However, the LifeLines-DEEP cohort data did not reveal any significant associations between *CrAssphage* and human disease conditions (Edwards et al., 2019). *CrAssphage* was a double-stranded DNA virus, currently without more detailed classification, had been identified as the most prevalent but poorly understood phage in the human gut, comprising almost 90% of sequences in the gut virome (Yutin et al., 2018). Recently, Shkoporov et al. confirmed the isolation of *CrAssphage* and found that this bacteriophage infected the *Bacteroides intestinalis*, and its proliferation was independent of bacteria (Shkoporov et al., 2018). Bacteriophage was an essential factor shaping the ecological homeostasis in the gut. Bacteriophage rarely encoded the antibiotic resistance genes and was also reported to be effective for some fatal infections (Enault et al., 2017; Zuo et al., 2017). The present study showed that the *CrAssphage* was more abundant in the healthy control than CRC, which indicated a promising potential treatment strategy for CRC through fecal *CrAssphage* transplantation in the future.

We also found that some bacteriophages were associated with genetic mutations. *Pseudomonas_phage_phiKZ* was associated with wild-type KRAS. *Pseudomonas* species contributed to the enterocyte’s apoptosis and the proliferation of intestinal stem cells, activating the carcinogenic pathway (Markou and Apidianakis, 2014). Bacteriophages could eliminate the carcinogenetic functions of bacteria in developing colorectal cancer *via* an enhanced immune system (Gogokhia et al., 2019; Stern et al., 2019). In addition, we also found *Lachnoclostridium phocaeense*, *Bifidobacterium breve*, and several phages such as *Bacillus_phage_PfEFR_5*, *Uncultured_phage_WW_nAnB_strain_2*, *Enterobacteria_phage_mEpX2* were found enriched in the CRC patients with MSI. Studies revealed that CRC patients with wild-type KRAS and MSI could benefit from targeted chemotherapy, which might be mediated by gut bacteria and phages described above (Nakatsu et al., 2018; Kannen et al., 2019; Zheng et al., 2019; Phipps et al., 2020). However, the detailed mechanisms still needed deeper investigation.

We then investigated the dynamic networks between bacteria and viruses at different stages of CRC. The gross relationship evaluation indicated that one microbe correlates with more than one virus in the gut, implying that the bacteria might be affected by several viruses, especially the bacteriophages. We found that some Firmicutes and Actinobacteria showed negative associations with *Salmonella* and *Escherichia* viruses but positive associations with some phages like *uncultured crAssphage* and *Enterobacteria phages*. And we also displayed that the *CrAssphage* was closely associated with butyrate-producing bacteria such as *Eubacterium rectale*, *Eubacterium cellulosolvens*, and *Butyrivibrio proteoclasticus* at different stages of CRC. This might indicate the co-expansion of bacteria and viruses and the prohibition roles of bacteriophages, suggesting the roles of bacteria-bacteriophage dynamic interactions in maintaining the hemostasis of the gut. In addition, when explored the bacteria-viruses interactions, we found that more viruses were involved in the network in CRC than healthy control and adenoma, which was consistent with the increased closeness centrality and decreased degree centrality in CRC. We also displayed that the anti-inflammatory *Faecalibacterium prausnitzii* was only negatively associated with *Parabacteroides_phage_YZ_2015a* at the CRC stage, indicating the dramatic changes in the microbial structure and relationships. Besides, we found that the *Escherichia virus* had increased relative abundance in adenoma and CRC and the essential connections in the adenoma and CRC networks. *Escherichia virus* was negatively associated with some butyrate-producing bacteria, indicating its potential carcinogenesis role in CRC development. We also found that the *Salmonella virus LSPA1* was negatively associated with *Bifidobacterium dentium* but positively associated with *Fusobacterium nucleatum*, a widely studied CRC promotion pathogen at the CRC stage. These relationships strongly suggested the candidate contributing role of *Salmonella virus LSPA1* in the development of CRC.

Despite the bacteria-virus relationships, the bacteria and viruses themselves also had complicated interactions. Genus *Fusobacterium* had been confirmed the causality with CRC, as well as *Parvimonas* and *Porphyromonas*. We revealed that these species also undertook essential roles in the network, as they showed close correlations with other microbes in adenoma and healthy control. Compared with bacteria, the network of viruses themselves was less close-knit. Most of the dominant critical members in the CRC were viruses, such as *Salmonella viruses*, *Escherichia viruses*, and *endogenous retrovirus*. Interestingly, recent evidence had demonstrated that endogenous retrovirus was associated with higher responding rates to cancer immunology therapy in renal cell carcinoma (Panda et al., 2018). The mechanism might be due to the activation of the immune checkpoints by increased immune infiltration. Of course, some harmful viruses were found to be able to deprive the beneficial functions of commensal bacteria (Kernbauer et al., 2014). Only one bacteriophage was identified as a critical member in adenoma, while as expected, *uncultured crAssphage* dominated in the healthy control group, followed by *Enterobacteria phages*.

The diagnostic test was performed based on bacteria and viruses and validated in independent cohorts in the present study. The AUROC of bacteria showed a better ability to detect CRC than viruses in an independent cohort, consistent with other studies (Nakatsu et al., 2018). We chose five microbes to establish the diagnostic panel and confirmed its relatively good discernibility of CRC from healthy controls. To get a higher possibility for detecting CRC, fifteen microbes, including bacteria and viruses, were determined and tested in the combined cohorts from Shanghai, Hong Kong, and Japan. The result showed a similar AUROC when compared with the five microbes panel.

In conclusion, we performed a metagenomics study of the three stages of CRC development. We confirmed the carcinogenetic pathogens and novel candidate microbes and viruses involved in the development of CRC. The dynamic and complicated network between gut bacteria and viruses was also evaluated to show the unique profile that changed at different stages of CRC. In the end, we developed the diagnostic panel, including bacteria and viruses for the detection of CRC. We validated it in independent cohorts, which enhance the reliability of gut microbiome-based studies. However, we did not have complete clinical test data in three groups, such as liver and kidney function, which needed to be amended in the future study. Our study provided reliable evidence for manipulating the gut microbiome for the diagnosis and treatment strategy of CRC.

## Data Availability Statement

The datasets presented in this study can be found in online repositories. The names of the repository/repositories and accession number(s) can be found below: NCBI BioProject, PRJNA706060 and PRJNA514108.

## Ethics Statement

The studies involving human participants were reviewed and approved by Ethics Committee of Shanghai Tenth People’s Hospital. The patients/participants provided their written informed consent to participate in this study.

## Author Contributions

HQ and RG take responsibility for the integrity of the work as a whole, from inception to published article. RG wrote this manuscript. RG, HL, and YZ performed the data check and analysis. YFZ, XZ, YL, HZ, KX, and RY helped collect all the subject information and samples. YZ, YFZ, XZ, CK, and CC performed the data visualization and interpretation of data. NQ performed the sample sequencing and analysis. RG and HQ designed and guided the whole study. All authors contributed to the article and approved the submitted version.

## Funding

This work was supported by grants from the National Natural Science Foundation of China (Nos. 81730102, 81230057, and 81472262); Emerging Cutting-Edge Technology Joint Research projects of Shanghai (No. SHDC12017112); Training program of the National Natural Science Foundation of China of Shanghai Tenth People’s Hospital (SYGZRPY2017024); “Climbing” plan of Shanghai Tenth People’s Hospital (2018SYPDRC030); the Fundamental Research Funds for the Central Universities (22120180575); and Special Project of Clinical Research on Health Care Industry of Shanghai Municipal Health Commission (20194Y0483).

## Conflict of Interest

The authors declare that the research was conducted in the absence of any commercial or financial relationships that could be construed as a potential conflict of interest.

## Supplementary Material

Supplementary Figure 1 The taxonomic alteration of the gut microbiome in healthy control (HC), adenoma, and colorectal cancer (CRC). **(A)** Alterations of the gut microbiome in HC, adenoma, and CRC groups at the phylum level. **(B) **The relative abundances of top 25 bacteria in HC, adenoma, and CRC groups at the species level. **(C)** The relative abundances of top 20 viruses in HC, adenoma, and CRC groups at the species level.

Supplementary Figure 2 Different dominated bacteria **(A)** and viruses **(B)** in colorectal cancer and healthy control, as well as bacteria **(C)** and viruses **(D)** differences at early stages (stage I+II) and later stages (stage III+IV) of colorectal cancer by LEfSe.

Supplementary Figure 3 Dominated gut microbiomes associated with gene mutations. **(A)** Gut bacteria dominated in the colorectal cancer samples with KRAS mutations or wild. **(B)** Gut bacteria associated with microsatellite stabilization (MSS) and microsatellite instabilization (MSI) in colorectal cancer. **(C)** Gut viruses associated with MSS and MSI in colorectal cancer.

Supplementary Figure 4 The correlations of the gut bacteria **(A)** and viruses **(B)** in healthy control, adenoma, and colorectal cancer.

Supplementary Figure 5 The indexes and function alteration in adenoma and colorectal cancer. The betweenness **(A)**, closeness **(B)**, and degrees **(C)** of the gut microbiome among the control (colored in green), adenoma (colored in amber), and CRC (colored in dubonnet) groups. Principal coordinate analysis of the functions change of gut microbiome at KO level **(D)** between adenoma and healthy control and that at pathway level between CRC and control **(E)**. **P<0.01 ***P<0.001

Supplementary Figure 6 The relationships of bacteria and viruses in all the samples. *P <0.05 + P <0.01.

Supplementary Figure 7 Bacteria and viruses selected for diagnostic models for colorectal cancer. **(A)**. The top 5 bacteria and viruses were chosen to diagnose colorectal cancer in Hong Kong and Japanese cohorts. **(B)**. Top 12 bacteria and viruses selected for the ROC of diagnosis of colorectal cancer in Hong Kong, Japanese, and Shanghai cohorts.

Supplementary Table 1 The characteristics of enrolled subjects in this study (Mean ± Standard Deviation). *One patient had two cancers in both ascending colon and rectum, which was counted in both ascending colon cancer and rectal cancer. A, Ascending colon; T, Transversal colon; D, Descending colon; S, Sigmoid colon; R, Rectum. ^#^Mean ± Standard error of the mean (SEM). WBC, White blood cell; RBC, Red blood cell; ALT, Alanine transaminase; AST, Aspartate aminotransferase; CEA, Carcinoembryonic antigen; AFP, Alpha-fetoprotein.

## Abbreviations

CRC, colorectal cancer; CRA, colorectal adenoma; AUROC, the area under the receiver operating characteristic curves; ORFs, Open reading frames; WBC, White blood cell; RBC, Red blood cell; ALT, Alanine transaminase; AST, Aspartate aminotransferase; CEA, Carcinoembryonic antigen; AFP, Alpha-fetoprotein.
