# Supplementary figures and images for: Alterations, Interactions, and Diagnostic Potential of Gut Bacteria and Viruses in Colorectal Cancer
Source: Front Cell Infect Microbiol. 2021 Jul 6;11:657867. doi: 10.3389/fcimb.2021.657867 (PMC8294192; doi:10.3389/fcimb.2021.657867)

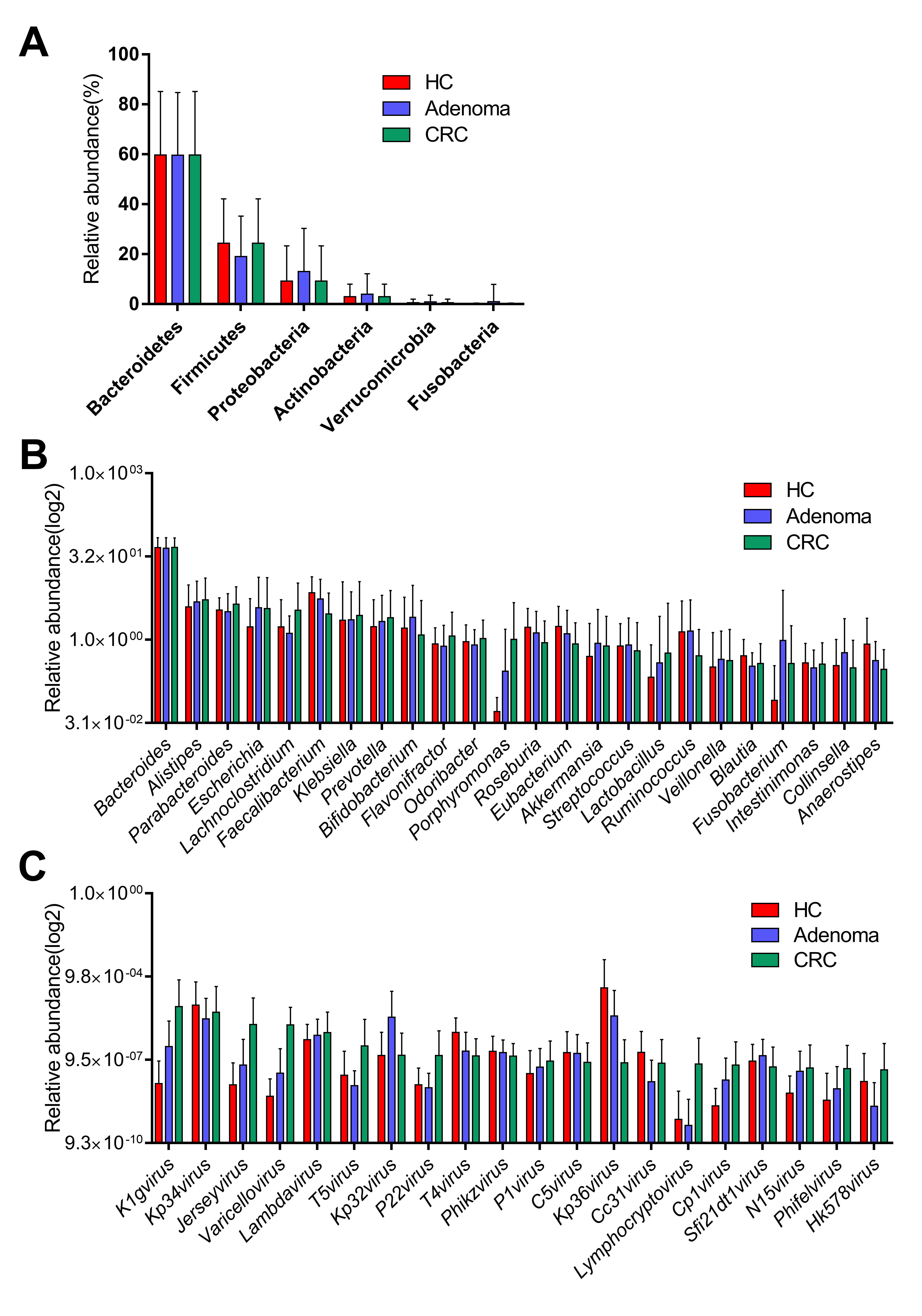

Supplement: Supplementary Figure 1 — The taxonomic alteration of the gut microbiome in healthy control (HC), adenoma, and colorectal cancer (CRC). (A) Alterations of the gut microbiome in HC, adenoma, and CRC groups at the phylum level. (B) The relative abundances of top 25 bacteria in HC, adenoma, and CRC groups at the species level. (C) The relative abundances of top 20 viruses in HC, adenoma, and CRC groups at the species level. [file Image_1.tif]

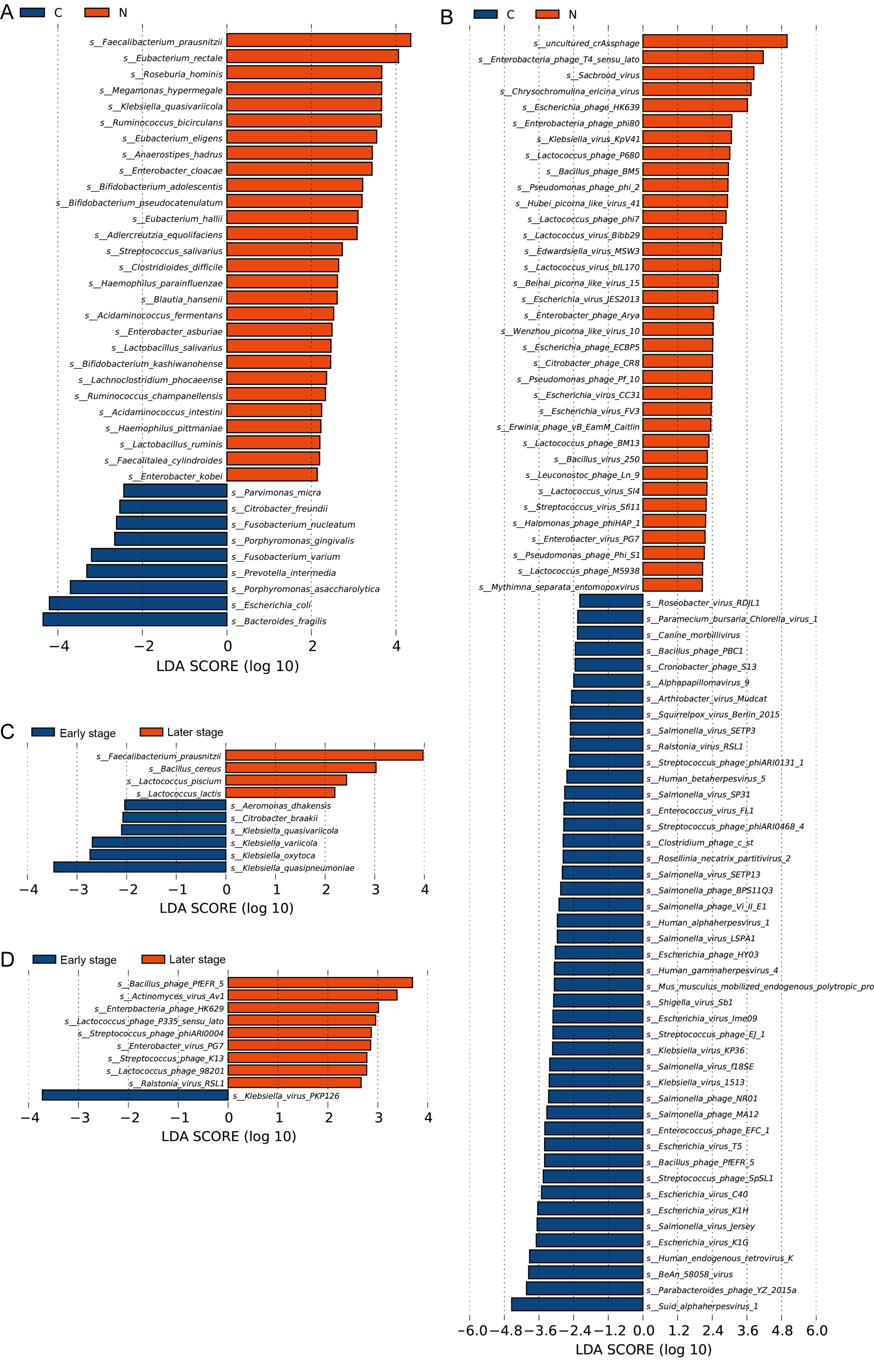

Supplement: Supplementary Figure 2 — Different dominated bacteria (A) and viruses (B) in colorectal cancer and healthy control, as well as bacteria (C) and viruses (D) differences at early stages (stage I+II) and later stages (stage III+IV) of colorectal cancer by LEfSe. [file Image_2.tif]

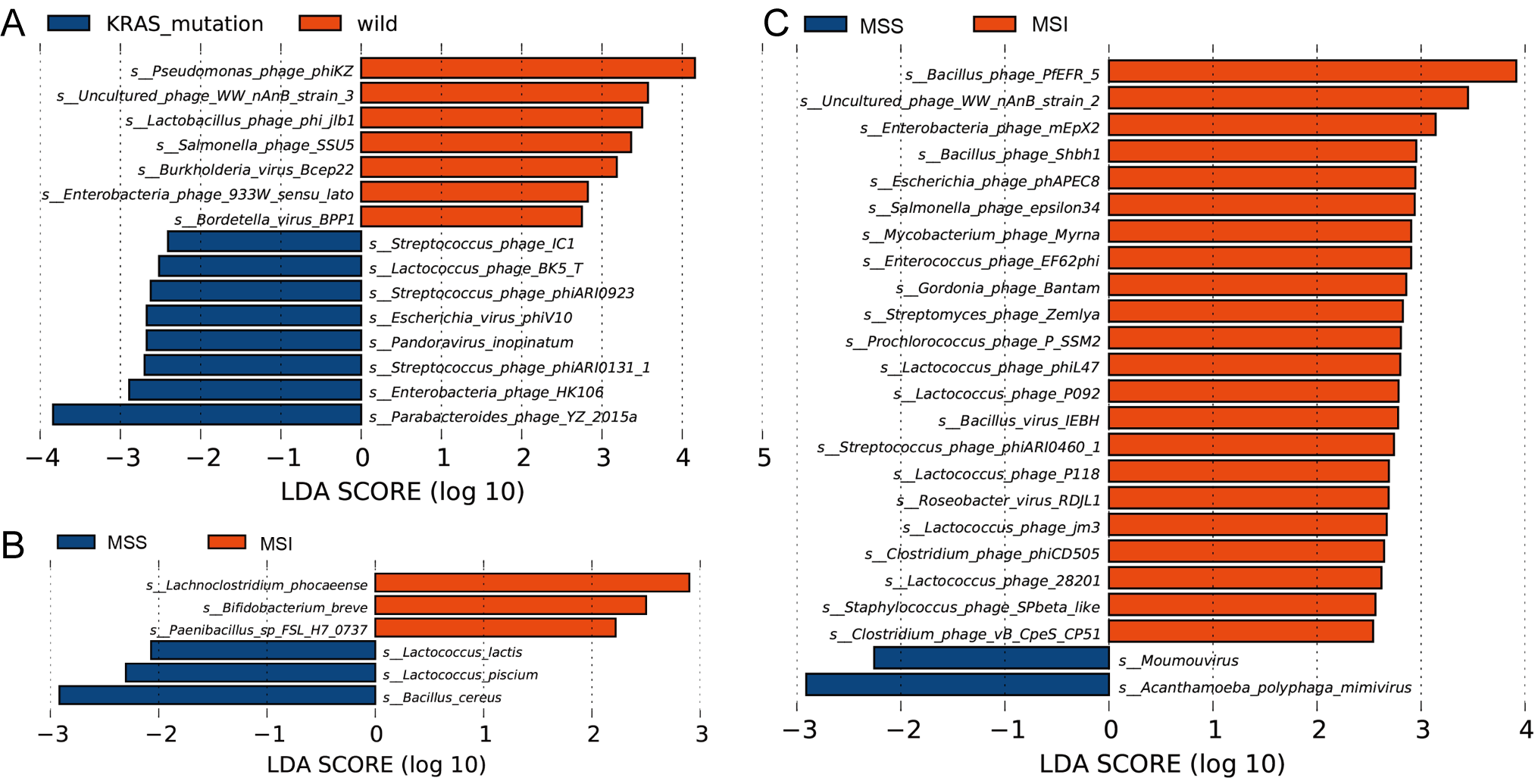

Supplement: Supplementary Figure 3 — Dominated gut microbiomes associated with gene mutations. (A) Gut bacteria dominated in the colorectal cancer samples with KRAS mutations or wild. (B) Gut bacteria associated with microsatellite stabilization (MSS) and microsatellite instabilization (MSI) in colorectal cancer. (C) Gut viruses associated with MSS and MSI in colorectal cancer. [file Image_3.tif]

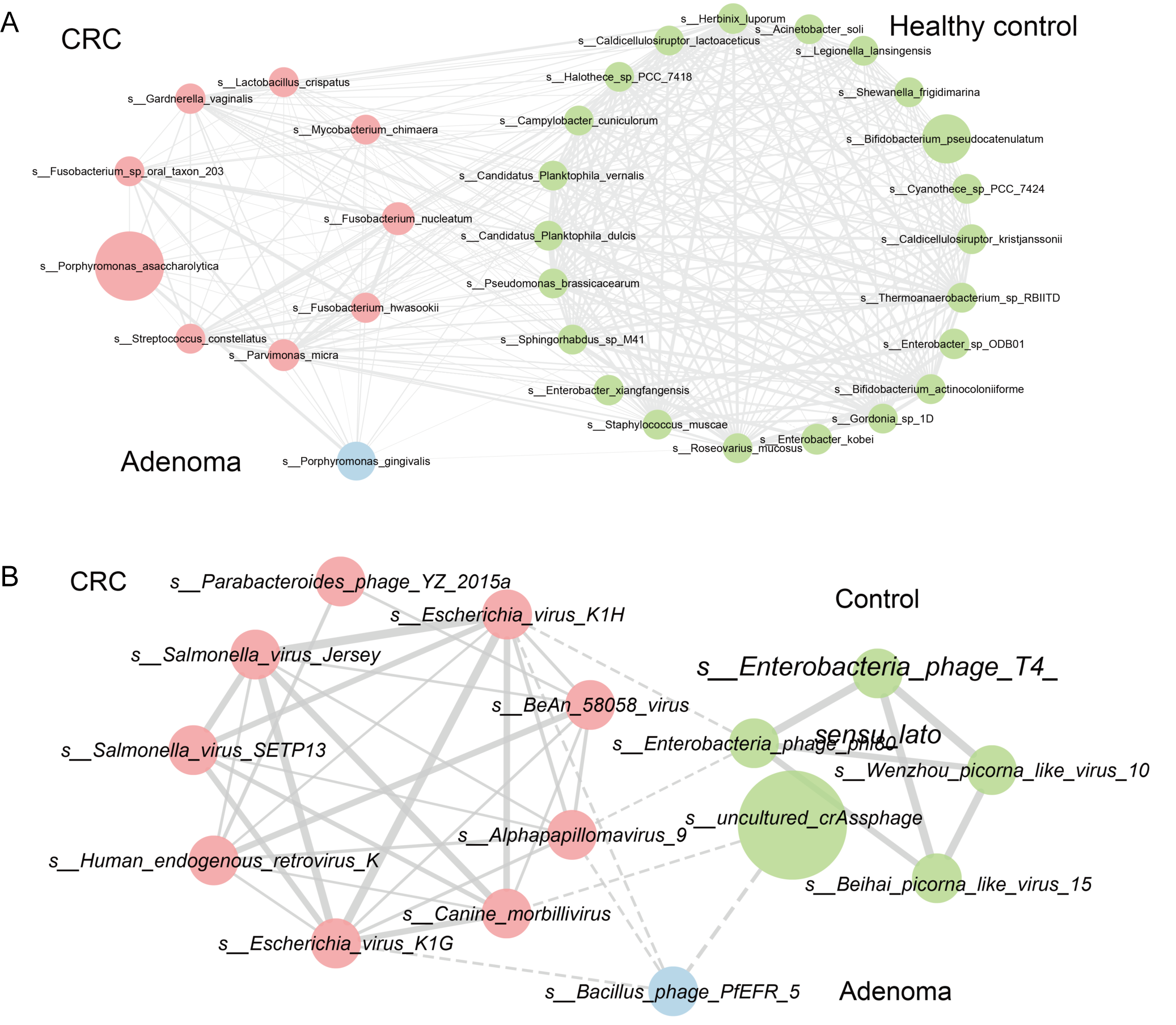

Supplement: Supplementary Figure 4 — The correlations of the gut bacteria (A) and viruses (B) in healthy control, adenoma, and colorectal cancer. [file Image_4.tif]

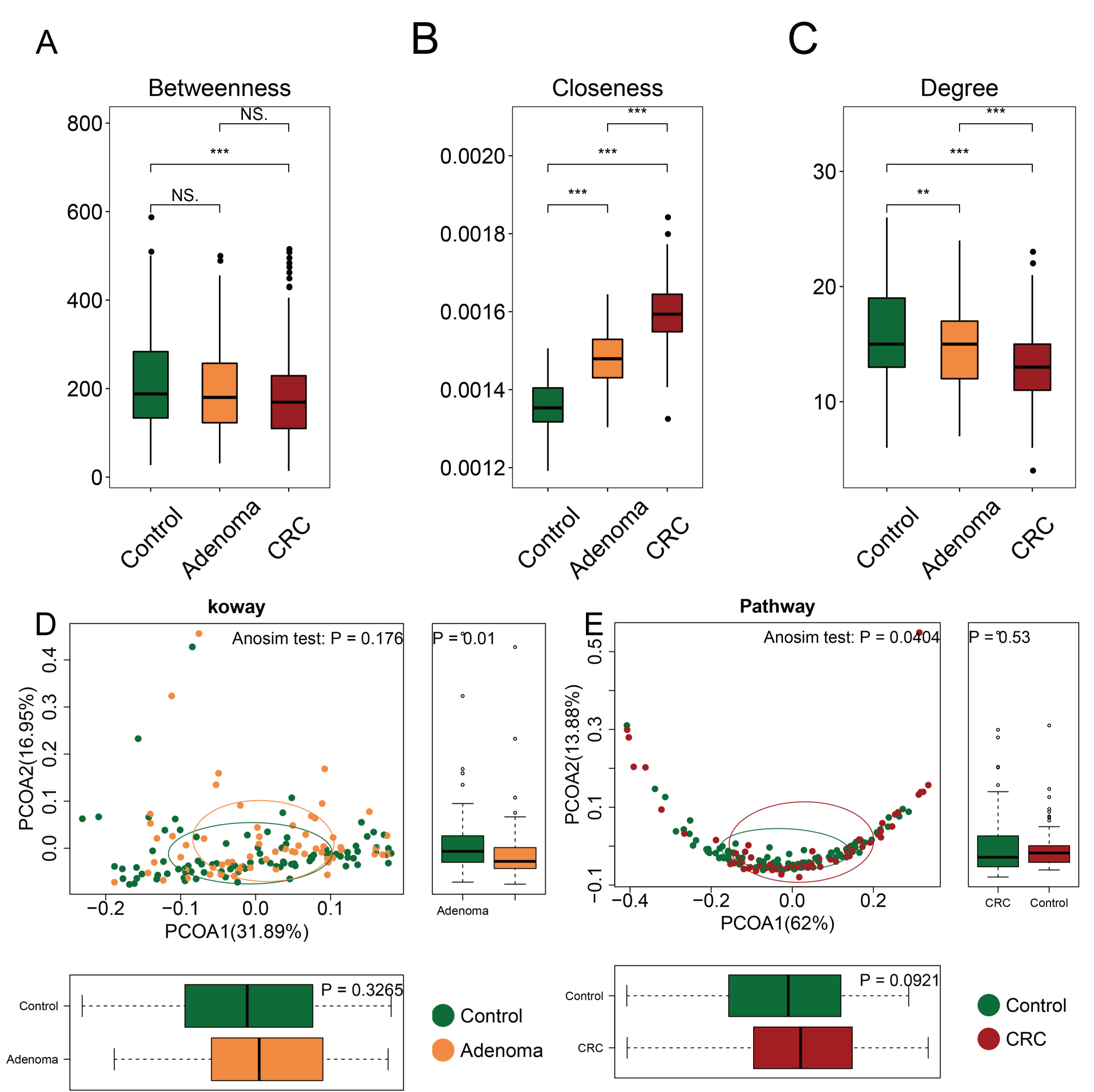

Supplement: Supplementary Figure 5 — The indexes and function alteration in adenoma and colorectal cancer. The betweenness (A), closeness (B), and degrees (C) of the gut microbiome among the control (colored in green), adenoma (colored in amber), and CRC (colored in dubonnet) groups. Principal coordinate analysis of the functions change of gut microbiome at KO level (D) between adenoma and healthy control and that at pathway level between CRC and control (E). **P<0.01 ***P<0.001 [file Image_5.tif]

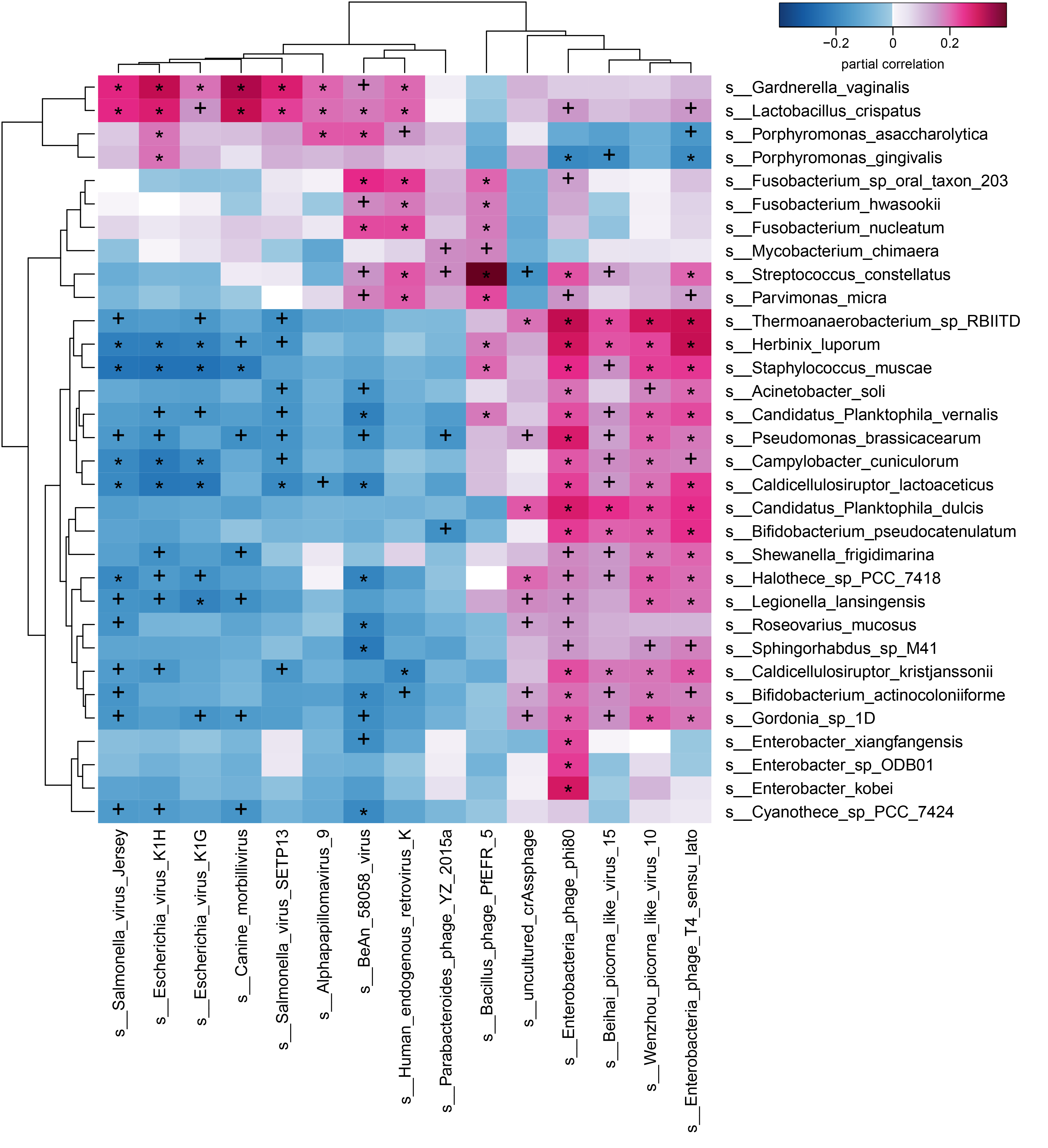

Supplement: Supplementary Figure 6 — The relationships of bacteria and viruses in all the samples. *P <0.05 + P <0.01. [file Image_6.tif]

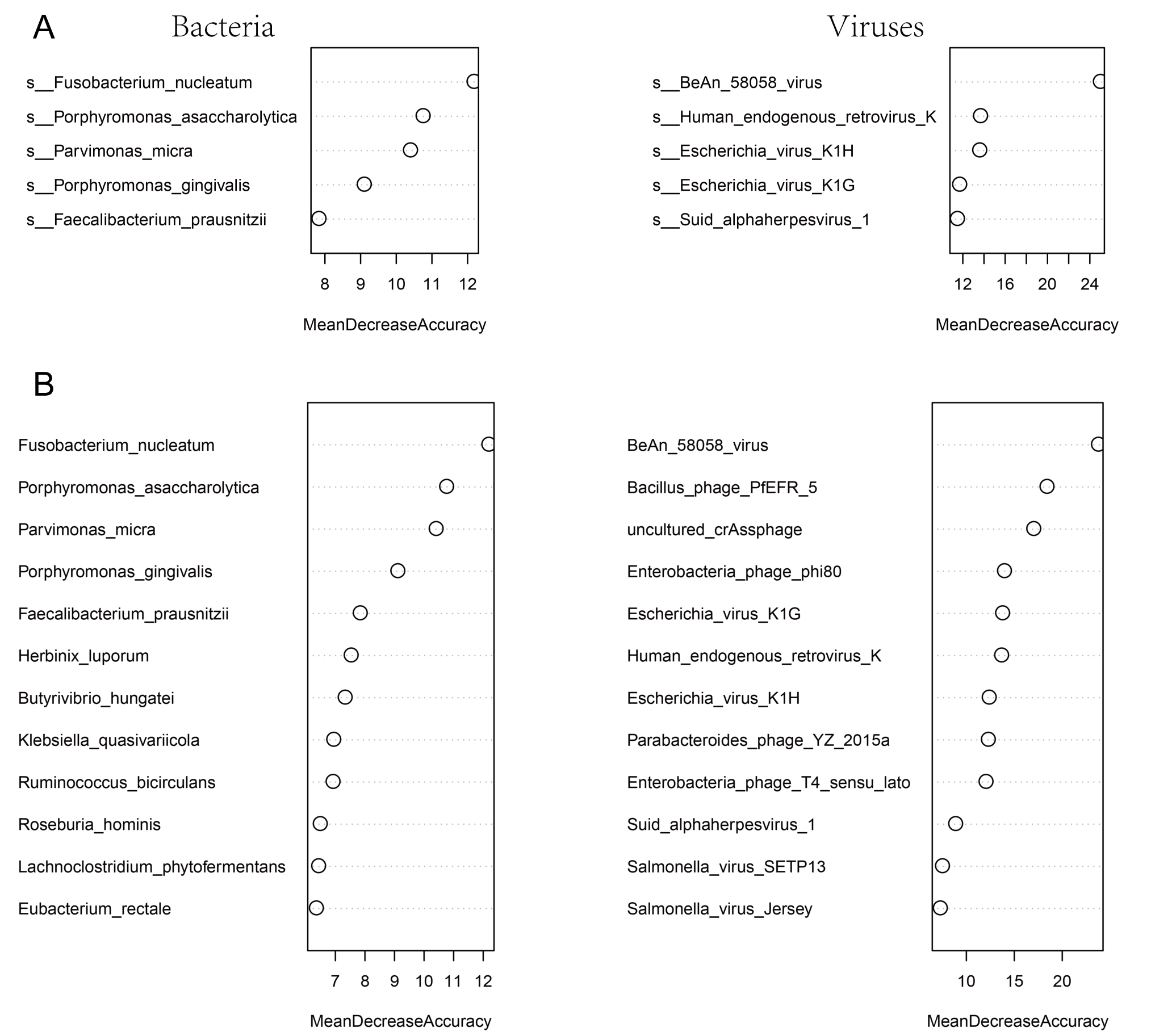

Supplement: Supplementary Figure 7 — Bacteria and viruses selected for diagnostic models for colorectal cancer. (A). The top 5 bacteria and viruses were chosen to diagnose colorectal cancer in Hong Kong and Japanese cohorts. (B). Top 12 bacteria and viruses selected for the ROC of diagnosis of colorectal cancer in Hong Kong, Japanese, and Shanghai cohorts. [file Image_7.tif]
