# Supplementary material for: Alterations, Interactions, and Diagnostic Potential of Gut Bacteria and Viruses in Colorectal Cancer
Source: Front Cell Infect Microbiol. 2021 Jul 6;11:657867. doi: 10.3389/fcimb.2021.657867 (PMC8294192; doi:10.3389/fcimb.2021.657867)
Supplement: Supplementary Table 1 — The characteristics of enrolled subjects in this study (Mean ± Standard Deviation). *One patient had two cancers in both ascending colon and rectum, which was counted in both ascending colon cancer and rectal cancer. A, Ascending colon; T, Transversal colon; D, Descending colon; S, Sigmoid colon; R, Rectum. #Mean ± Standard error of the mean (SEM). WBC, White blood cell; RBC, Red blood cell; ALT, Alanine transaminase; AST, Aspartate aminotransferase; CEA, Carcinoembryonic antigen; AFP, Alpha-fetoprotein. [file Table_1.docx]

|  | Colorectal cancer | Adenoma | Healthy controls | P value |
| --- | --- | --- | --- | --- |
| No. of individuals | 71 | 63 | 91 |  |
| Male (%) | 42(59.15) | 34(53.97) | 38(41.76) | 0.07 |
| Age(years) | 61.85±10.96 | 63.22±7.29 | 60.23±5.06 | 0.06 |
| BMI | 22.96±3.85 | 23.81±3.03 | 23.32±1.97 | 0.11 |
| A/T/D/S/R* | 16/5/2/8/40 | / | / | / |
| Stage I/II/III/IV | 9/30/27/5 | / | / | / |
| KRAS mutation (%) | 35(49.3%) | / | / | / |
| Fecal sampling before and after colonoscopy (before: after) | 43:28 | 28:35 | 40:51 | 0.07 |
| WBC (×10^9^/L) | 6.20±2.27 | 5.75±2.09 | 5.81±2.03$ | 0.36 |
| RBC(×10^12^/L) | 4.34±0.84 | 4.59±1.24 | 4.75±1.62$ | 0.14 |
| Hemoglobin(g/L) | 119.00±22.95 | 115.45±21.37 | 120.76±25.93$ | 0.39 |
| Platelet(×10^9^/L) | 262.80±110.40 | 289.35±121.70 | 276.84±115.43$ | 0.41 |
| Albumin(g/L) | 39.28±6.02 | / | / | / |
| Globulinemia(g/L) | 26.85±4.04 | / | / | / |
| Total bile acid (umol/L) | 8.84±5.89 | / | / | / |
| Conjugatedbilirubin(umol/L) | 2.95±1.44 | / | / | / |
| Glucose(mmol/L) | 5.66±1.79 | / | / | / |
| ALT(U/L) | 15.1±9.00 | / | / | / |
| AST(U/L) | 18.33±7.84 | / | / | / |
| CEA(ng/ml)# | 25.05±14.52 | / | / | / |
| AFP(ng/ml) | 2.99±1.22 | / | / | / |
| CA153(U/ml) | 10.37±4.66 | / | / | / |
| CA-125(U/ml) | 16.98±19.91 | / | / | / |
| CA199(U/ml)# | 34.59±15.38 | / | / | / |
| CA724(U/ml)# | 6.68±1.66 | / | / | / |
| CA50(IU/ml)# | 16.30±5.61 | / | / | / |

**Supplementary table 1** The characteristics of enrolled subjects in this study (Mean ± Standard Deviation)

* One patient had two cancers in both ascending colon and rectum, which was counted in both ascending colon cancer and rectal cancer

A: Ascending colon T: Transversal colon D: Descending colon S: Sigmoid colon R: Rectum

# Mean±Standard error of mean (SEM)

$ n=30 for healthy controls

WBC: White blood cell; RBC:Red blood cell; ALT: Alanine transaminase; AST: Aspartate aminotransferase; CEA: Carcinoembryonic antigen; AFP: Alpha-fetoprotein.
